# North Equatorial Current and Kuroshio velocity variations affect body length and distribution of the Japanese eel *Anguilla japonica* in Taiwan and Japan

**DOI:** 10.1038/s41598-022-06669-8

**Published:** 2022-02-21

**Authors:** Kuan-Mei Hsiung, Yi-Chun Kuo, Yen-Ting Lin, Yu-Heng Tseng, Yu-San Han

**Affiliations:** 1 Institute of Oceanography, National Taiwan University, No. 1, Sec. 4, Roosevelt Road, Taipei, 10617 Taiwan, ROC; 2 Institute of Fisheries Science, College of Life Science, National Taiwan University, Taipei, 10617 Taiwan, ROC; 3 School of Oceanography, Shanghai Jiao Tong University, No.1954 Huashan Road, Shanghai, 200030 China; 4 Graduate School of Frontier Sciences/Atmosphere and Ocean Research Institute, University of Toyko, 5-1-5, Kashiwanoha, Kashiwa, Chiba 277-8564 Japan

**Keywords:** Marine biology, Physical oceanography

## Abstract

The larval stage of Japanese eel travels a substantial distance over a long duration through the North Equatorial Current (NEC) and the Kuroshio, and the spawning behavior of mature eels leads to monthly arrival waves in eastern Taiwan between November and February. The total length (TL) of the glass eel relates to its larval duration and age; therefore, the TL can indicate the larval duration. The monthly mean TLs of eels along eastern Taiwan from 2010 to 2021 were used to estimate the batch age, and the recruitment patterns and relative abundances were compared. The TLs of glass eels followed a normal distribution, and the estimated ages were highly correlated with their mean TLs. Early recruit TLs were significantly greater than those of late recruits. The mean tracer drift time was longer in early recruitment months (November–December) than in later dates (February–March). The recruitment lag between Taiwan and Japan was approximately 1–1.5 months, with relative more abundance in Taiwan for the early recruits and in Japan for the late recruits. Speculated cohorts followed the main streams of the NEC and Kuroshio, and the monthly velocity changes of these currents could affect the mean TLs as well as the distribution patterns of Japanese glass eels in Taiwan and Japan.

## Introduction

The Japanese eel *Anguilla japonica* Temminck and Schlegel 1847 is a temperate catadromous fish with a complex life history that is mainly distributed in Taiwan, China, Korea, and Japan^1–5^. Although it is a commercially valuable aquaculture species in East Asia, large-scale commercial artificial propagation is currently not possible, and the fry used in aquaculture can only be obtained by capture in estuarine and coastal waters during their upstream migration. Japanese eel resources have been declining significantly since the late 1970s^6–8^ by capture of wild specimens, and the annual catch of adult wild Japanese eels in Japan has decreased from approximately 3000 tons in the 1970s to less than 80 tons in recent years (Annual Report of the Ministry of Agriculture, Forestry and Fisheries, Japan, https://www.maff.go.jp/j/tokei/kouhyou/naisui_gyosei/index.html). In response to this resource crisis, the Japanese eel was listed as an “endangered” species in 2013 by the Ministry of the Environment, Government of Japan. In 2014, it was included in the IUCN Red List of Threatened Species as an endangered species^9,10^, and in 2017, it was deemed “critically endangered” in the freshwater Red List by the Forest Bureau of the Council of Agriculture, Taiwan. A combination of factors has caused this decline, including habitat degradation, overfishing, and fluctuations in oceanic conditions^8,11,12^. To ensure the resource sustainability of the Japanese eel, a better understanding of its life history is necessary.

Mature eels spawn in the western waters of the West Mariana Ridge located at 12.5–16° N, 141–142° E within the North Equatorial Current (NEC)^2,3,13^ mainly between May and September^5,7,14,15^. The spawning event occurs at a depth of approximately 160–250 m^16^. After hatching, the *A. japonica* larvae (leptocephali) have a limited swimming speed, while the current velocity of the surrounding NEC water is much greater (typical zonal velocity 30 cm/s)^17^. Thus, the larvae drift passively by way of oceanic currents for long-distance dispersal. In addition, as the larvae drift with the current, they remain in the upper surface waters (approximately 50 m deep) during the daytime and dive into deeper waters (approximately 150 m deep) at night^18^, which is known as diel vertical migration (DVM). The NEC bifurcates at its westernmost boundary off the coast of the Philippines into the north-flowing Kuroshio and south-flowing Mindanao currents, a feature known as the NEC bifurcation^19^. *A. japonica* larvae must enter the Kuroshio in the bifurcation zone to reach their habitats in East Asian countries^13^. The leptocephali metamorphose into juvenile eels (glass eels) after 4–6 months of drifting^2,3,20,21^. Metamorphosing larvae (52.7–61.2 mm) were collected only in the area to the east of Taiwan (21–26° N, 121–129° E), while glass eels (51.3–61.2 mm) were found only within or west of the Kuroshio^13^. These distributions suggest that leptocephali begin to metamorphose within or just east of the Kuroshio. After completion of metamorphosis, the glass eels adopt a benthic sheltering behavior to escape he oceanic current and actively swim toward nearby estuaries and rivers for growth^1^. Generally, the early life stages of *A. japonica* in the ocean are the most vulnerable periods. Biological and physical changes in the oceanic environment may significantly influence their transport processes, mortality, growth rates, and recruitment dynamics^11,22–24^.

**Figure 1:**
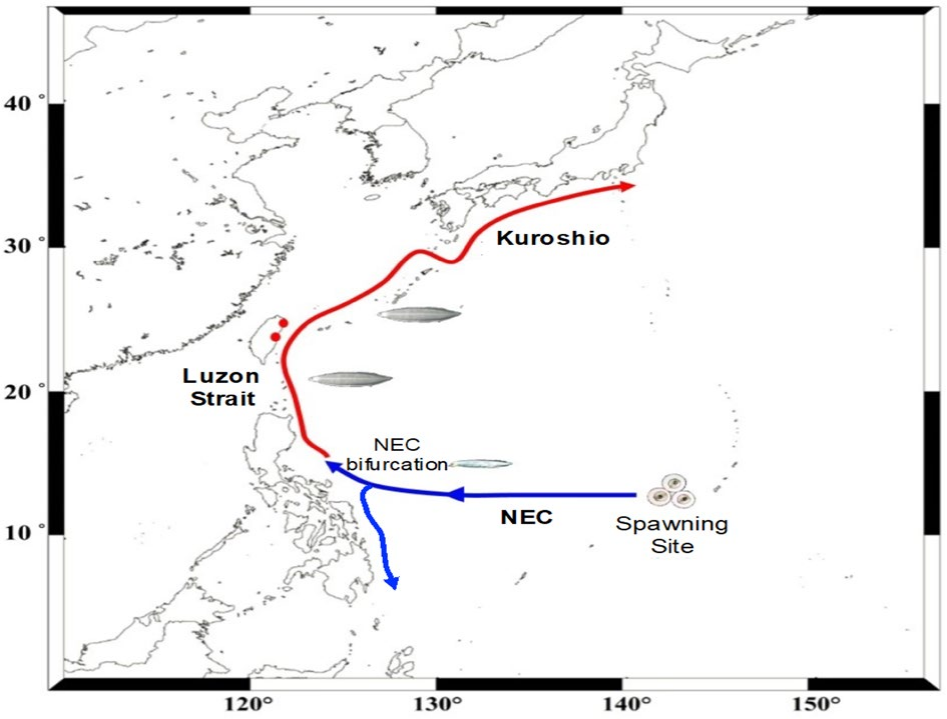
Map showing the spawning site and transport route of the eel larvae to Taiwan and Japan. Red dots indicate the sampling sites of the Japanese glass eel (Yilan and Hualien).

Tsukamoto et al.^25^ analyzed the otolith microstructure of *A. japonica* leptocephali collected near the spawning area in July 1991 and estimated their ages to determine spawning times. These leptocephali had a total length of 10–30 mm and consisted of two batches of individuals hatched during the new moon periods of May and June. The “New Moon Hypothesis” was proposed to explain the timing of eel spawning^4^. This hypothesis assumes that the abundance of recruited glass eels along their transport route should be uneven and may exhibit batch-like cohorts. Among East Asian countries, Taiwan is closest to the spawning ground of the Japanese eel, and it is the first country to capture this species (Fig. 1). Batch-like waves were indeed found in the waters of eastern Taiwan, where the main stream of the Kuroshio flows along its east coast. The arrival waves have a periodicity of approximately 1 month, which may be in line with the hypothesized monthly spawning behavior during the new moon, coincides with their monthly spawning behavior^21^.

In a previous study, the *A. japonica* glass eel specimens collected from Taiwan from 1984 to 2013 indicated that the mean total length (TL) and larval duration (LD) in three climates (El Niño, normal, and La Niña) were significantly different, with El Niño years having larger TLs and LDs than those of the La Niña years^26^. This finding suggests that El Niño-Southern Oscillation (ENSO) events may have significant impacts on the TL and LD of the glass eel, since they are connected with NEC bifurcation^27,28^, current transport^29^, and current velocity^30^. The eel spawning site and passive transport by the NEC may affect the fluctuation of TL and LD in the glass eel^26^. If the eel spawns south of the salinity front of the NEC, the southward movement of the salinity front associated with the northward movement of the NEC bifurcation during El Niño years may cause the larvae to experience slower currents and broaden the distance between their spawning ground and NEC bifurcation. Thus, the time needed for eel larvae to enter the Kuroshio from their spawning grounds would be prolonged. In addition, according to the previous study, rearing experiments showed that the TLs of leptocephali decreased by an average of 12.5% during metamorphosis^31^. We assumed that most of the leptocephali shrink in a similar ratio, so it is reasonable to assume that TLs of glass eels correlate with the age of glass eels. Therefore, TL could be an alternate for representing the LD measure. NEC/Kuroshio velocity variations are monthly dependent^32^; therefore, glass eels sampled from different months may show fluctuations of age as well as TL.

According to Tsukamoto et al.^25^, the analysis results of the otolith microstructure of *A. japonica* leptocephali were collected near the spawning area in July 1991 and estimated their age in order to determine the spawning times of *A. japonica*. These leptocephali had a total length of 10–30 mm and consisted of individuals hatched during the new moon periods of May and June. The “New Moon Hypothesis,” therefore was proposed to explain the timing of eel spawning. Furthermore, Han et al.^21^ found batch-like waves of glass eel arrival in the Yilan offshore area, with peaks occurring between the last quarter and first quarter lunar periods, and one-month periodicity. The results from Han et al.^21^ seem to correspond logically with the New Moon Hypothesis. Additionally, among the East Asian countries, Yilan, Taiwan is closest to the spawning ground of the Japanese eel, and it is the first place to receive recruitment waves of *A. japonica*. Therefore, the individual variation in the daily age should be most insignificant, and the dynamics of glass eel arrivals in the Yilan offshore area may serve as a proper assessment of monthly larval cohorts from the spawning ground because most glass eels in Yilan are caught by offshore ships (more than 70%), which are not affected by tidal stream^21^ and thus could better reflect the real recruitment dynamics of the glass eel.

To better understand the effects of monthly dependent NEC/Kuroshio velocity variations on eel larval size in Taiwan, as well as its distribution pattern in East Asia, the TL of *A. japonica* glass eel in Taiwan and its recruitment dynamics in Taiwan and Japan were analyzed. The estimated transport time of each recruitment cohort was calculated using the glass eel arrival time and the nearest new moon period six month prior. A particle tracking simulation was used to estimate the transport patterns of the eel larvae spawned between May and September from the supposed spawning ground to Taiwan.

## Materials and methods

### Glass eel collection and total length (TL) measurement

The estuaries of the Yilan and Hualien counties of eastern Taiwan were the chosen regions for monitoring the arrival of Japanese glass eels (Fig. 1). This species is easily distinguished from other eels in the glass eel stage (e.g., *A. marmorata*, *A. bicolor pacifica*, and *A. luzonensis*) by their lack of caudal cutaneous pigmentation^33^, and they are the target eel species for Taiwanese fisheries in winter. The pigmentation stage of the glass eel (V_A_, V_B_, VI_A1_, VI_A2_, VI_A3_, VI_A4_, and VI_B_) was determined based on Tesch^1^. The main fishing seasons in Taiwan and Japan are from November to February and December to March, respectively. The Japanese glass eel specimens were collected at night during the fishing season using a hand-trawling net and a fyke net in Japan and mainly using a boat net in Taiwan. They were collected from Yilan (24.7163° N, 121.8348° E) and Hualien (23.4612° N, 121.5008° E) in Taiwan between December 2010 and February 2021 and from Kochi (33.3335° N, 133.3152° E), Chiba (35.3626° N, 140.0623° E), Aichi (35.1043° N, 136.5450° E) and Tanegashima (30.3426° N, 130.5852° E) in Japan between November 2010 and April 2018. A total of 5595 glass eels from Taiwan and 308 glass eels from Japan were used to measure the monthly TLs.

All of the Japanese glass eel samples were fixed and preserved in 95% ethanol after collection. The samples were measured after 1 month when the shrinkage of the samples stopped (approximately 6%, data not shown). Older eels may stay longer in estuaries, and their growth may be markedly affected by the estuarine environment. Thus, only glass eels in the pigment stages V_A_ and V_B_, which are considered the earliest phases of glass eels arriving in estuaries^1^, and should be less affected by feeding and environmental conditions and suit to be used to compare the differences of the TL in the glass eel stage. Glass eels in the pigment stages V_A_ and V_B_ were selected in this study and their TL was measured to the nearest 0.1 mm.

This study assumed that the TL of the glass eel would be correlated with their age. If so, the TL and estimated age of the glass eel would also perform a positive correlation. Therefore, the linear regression between TL and estimated age has further been done.

Eel sampling was approved by the Fishery Agency, Council of Agriculture, Executive Yuan, Taiwan. Animal use approval “NTU-108-EL-00130” was obtained from the Animal Research Committee of National Taiwan University and reviewed by the Institutional Animal Care and Use Committee (IACUC).

### Glass eel catch information in Taiwan and Japan

The weekly/monthly observation catch data of the glass eel from 2010 through 2021 in Taiwan and Japan were collected by the Japan Aquaculture Information News (daily fax), the Taiwan Japanese Glass Eel Reporting System (Fisheries Agency, Council of Agriculture, Executive Yuan, Taiwan), and glass eel traders in Taiwan. The catch data included glass eels caught throughout Taiwan and Japan.

### Estimating the mean ages of the arrival batches recruiting to eastern Taiwan

Yilan and Hualien in Taiwan are the first locations to collect glass eels in Taiwan and East Asia, and the recruited samples showed arrival waves at approximately 1-month intervals^21^. Based on the weekly data from the Taiwan Japanese Glass Eel Reporting System and the daily information from the Japan Aquaculture Information News, the first arrival time of the Japanese glass eel in Yilan usually begins in mid-November. By assuming the eels spawn near the new moon period, and the mean ages of the glass eels from Taiwan were approximately 6 months^5,20,21^, the estimated spawning date of each arrival batch would have occurred during the nearest new moon approximately 6-month prior to the catch date. The estimated theoretical age (day) of each arrival batch of the glass eel was determined based on the date of its supposed spawning new moon and the date of its arrival peak in Yilan. Furthermore, for each arrival batch, at least 50 individuals were randomly chosen and their TLs were measured to assess the relationship between TL and the estimated age of Japanese glass eels.

### Analysis of the NEC/Kuroshio long-term monthly velocity

The Kuroshio is the main western boundary current in the North Pacific, and NEC is its upstream source. The westward flow speed of the NEC gradually increases toward the main stream. The NEC bifurcates before reaching the coast of the Philippines, while the northern branch develops into the Kuroshio. The northward Kuroshio is enhanced to the east of the Philippines and develops more baroclinity. Both the NEC and Kuroshio exhibit strong seasonal variability with clear monthly-dependent changes^34,35^. The surface climatological NEC bifurcation latitude is approximately 12.7° N based on 40 years of Simple Ocean Data Assimilation (SODA) reanalysis^36^. The bifurcation latitude moves toward the north with depth owing to the baroclinity of the NEC. The climatological mean of the subsurface (96 m) NEC bifurcation latitude was estimated to be approximately 13.6° N by Meng et al.^36^, which is similar to the estimation of 14° N using the 25-yr HYCOM reanalysis in this study. The seasonal variability of the bifurcation latitude is significant, reaching the southernmost latitude in May–June, and then moving northward until fall (October–November)^28,37,38^. The NEC main stream migrates meridionally along with the NEC bifurcation latitude. However, different segments of the NEC do not move simultaneously; hence, the time–space variations are more complex.

Considering the strong seasonal variability of the velocity change and the months that *A.japonica* pass through the NEC and Kuroshio, we analyzed the mainstream velocity (m/s) of NEC over 1994–2019 in the region of 13–14° N, 15–16° N and that of Kuroshio. We note that the traveling region of Japanese eel is within a mesoscale eddy-rich domain (122–170° E, 12–28° N^39^) of northwestern subtropical Pacific Ocean. Mesoscale eddies are mainly generated due to the instability of the STCC-NEC system^28,40^. While the tracking particles inevitably enter an anticyclonic eddy, their velocity will increase/decrease if the eddy center is to its north/south. For those entering a cyclonic eddy, the result is opposite. Therefore, we remove eddy influences to ensure a smoother NEC transport as follows:

1 $$\overline{{u}^{*}}=\overline{u}-\left(sign\left(\frac{-dSSHA}{dy}\right)\times \overline{\underset{l{\mathit{at}}_{i}<\mathit{lat}<l{\mathit{at}}_{i+1}}{\mathrm{max}}\left(v\right)}-\frac{\sum_{A}V}{A}\right) ,$$

where $u \,and\, v$ are the daily zonal and meridional velocity, respectively. $\overline{x}$ denotes the monthly mean of $x. A$ is the area bound by $l{at}_{i}-l{at}_{i+1}$ and $l{on}_{j}-l{on}_{j+1}$ (the longitude and latitude sections in Fig. 5). If the meridional SSHA gradient > 0 (< 0), the anomalous geostrophic current possibly due to a mesoscale eddy is westward (eastward). We remove the rotational speed by assuming the fluid motion is approximately solid-body rotation^41^. That is, the maximum meridional speed is equal to the maximum zonal speed. Then we exclude the NEC meandering, defined as $\frac{\sum_{A}V}{A}$.

For the Kuroshio region, the u and v in Eq. (1) are switched:

2 $$\overline{{v}^{*}}=\overline{v}-\left(sign\left(\frac{dSSHA}{dx}\right)\times \overline{\underset{\mathit{lon}<\mathit{lon}<l{\mathit{on}}_{1}}{\mathrm{max}}\left(u\right)}-\frac{\sum_{A}u}{A}\right).$$

### Ocean circulation model of the North Pacific

We conducted a Lagrangian tracer study based on the 1/12° global HYbrid Coordinate Ocean Model (HYCOM) (GLBv0.08, https://www.hycom.org/dataserver/gofs-3pt1/reanalysis, for 1994–2015 and GLBy0.08, https://www.hycom.org/dataserver/gofs-3pt1/analysis, for 2016–2019). The high-resolution HYCOM provides an appropriate modelling framework to simulate ocean circulation in the North Pacific, which is useful for investigating glass eel larval migration. HYCOM data are daily assimilative global results using three types of vertical coordinates: z-level, terrain-following, and isopycnic^42^. The model uses 32 vertical levels with a horizontal resolution of approximately 7 km. Quality controlled bathymetric data from the Naval Research Laboratory’s Digital Bathymetry Data Base 2 (NRL DBDB2) were used. Surface forces, including wind stress, wind speed, heat flux, and precipitation, were retrieved from the Navy Operational Global Atmospheric Prediction System (NOGAPS).

### Simulation of migration time from spawning ground to Luzon Strait

Tracer simulations were performed to study the migratory behavior of the larvae. Since the 1/12° global HYCOM reanalysis assimilated all available oceanic data, it reproduced the realistic flow patterns of the Kuroshio east of Taiwan and around the Luzon Strait^43^. An offline Lagrangian particle-tracking method was used to investigate the potential migration routes of eel larvae from the spawning area to the Luzon Strait (Fig. 1). Both ocean currents and biological swimming speeds were considered in the model. The former was extracted from the 1/12° global HYCOM climatology (1994–2019) and the latter increased linearly with age.

The representative tracer experiment was performed at a depth of 100 m from May to September, during the main spawning season of *A. japonica*, from 137–140° E to 12–16° N. The simulations have conducted with a setting at a depth of 50 m, 100 m, 150 m and DVM of 50–150 m, and the simulated transport pathways and times showed very similar patterns, suggesting the results may not be very sensitive to the DVM and chosen depth. Therefore, the average one, which was the depth at 100 m was selected in this study. Furthermore, a two-stage migration behavior of eel larvae is performed here^44^: 1. A flow-carried migration stage; 2. An active swimming migration stage. In the first stage, the eel moved passively by the transit of the water masses. In the second stage, an active swimming migration adds to the flow-carried migration. The overall mean tracer drift time from the presumed spawning site to Luzon Strait between 1994 and 2020 was calculated. The particles were set to drift passively in the first 10 days, and then swam actively afterward, i.e., grow to the leptocephali stage^22^. A total of 10,000 particles (0.5° × 0.5°) were released, starting from the first day of model month during May to September”.

The horizontal swimming direction of the eel larvae was then calculated along the direction of the ocean current^45^, defined as

3 $${u}_{s}=V\times \frac{{u}_{c}}{\sqrt{{u}_{c}^{2}+{v}_{c}^{2}}} , {v}_{s}=V\times \frac{{v}_{c}}{\sqrt{{u}_{c}^{2}+{v}_{c}^{2}}}.$$

Here, *u*_*s*_ and *v*_*s*_ represent the x and y components of the particle swimming velocity with the current, respectively. *V* is the eel larval swimming speed, and *u*_*c*_ and *v*_*c*_ are the x and y components of the current velocity, respectively. After hatching, the particles had a velocity of 1.5 cm/s from the 11th day and increased their speed by 1.5 cm/s per month up to 6 cm/s. The speed was maintained at 6 cm/s until the end of the simulation. A similar procedure was applied by Rypina et al.^46^ and Chang et al.^22^ to estimate the migration of American eels from the Sargasso Sea and Japanese eels from their spawning site in the Western Pacific Ocean. Only the particles with migration routes into the Kuroshio zone (18–20° N, 121–124° E) were considered effective, and those remaining in the western North Pacific Ocean, those entering the Mindanao Current, and those reaching the Kuroshio after 180 days were excluded. Therefore, only the particles entering into the Kuroshio zone within 180 days, the expected average age of the juvenile eels with successful migration, were considered.

### Data analysis

Differences in the total length (mean ± SD) between monthly samples were tested using a one-way analysis of variance (ANOVA) followed by Tukey’s honestly significant difference (HSD) multiple-comparison test. The data of recruitment percentage of Taiwan among months did not pass the normal distribution test; thus, nonparametric statistics of the Mann–Whitney test were performed to compare differences in the recruitment percentage of Taiwan among months. Linear regression was used to analyze the relationship between the total length and the estimated age of the glass eels. SPSS (Statistical Package for the Social Science) software (version 16.0) was used for the statistical analysis. Differences were considered significant at *p* < 0.05.

## Results

### Glass eel TL

**Figure 2:**
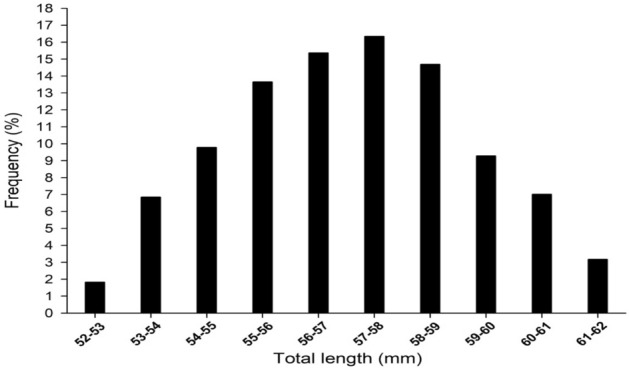
The frequency distribution map of the Japanese glass eel from Taiwan.

The Japanese glass eel specimens analyzed in this study were collected in the estuaries of Yilan and Hualien from November 2010 to February 2021. A total of 5595 specimens from Taiwan and 308 specimens of the Japanese glass eel from Japan were collected, and their TL was measured. The results showed the mean total length of the glass eels in Japan was 57.3 ± 2.0 mm (s.d.) which was greater than that of glass eel in Taiwan (56.8 ± 2.2 mm) (s.d.) with significance (*p* < 0.05). The TL frequency distribution ranged from 52 to 62 mm with an interval of 1 mm, forming a normal distribution (Fig. 2).

### The correlation of TL and estimated age of glass eels in eastern Taiwan

**Figure 3:**
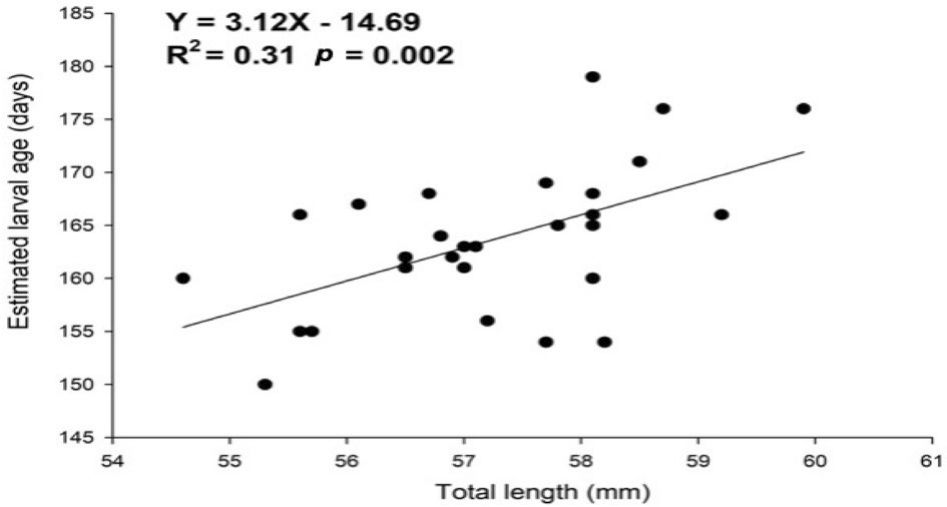
The regression between total length and estimated age of Japanese glass eels transported to eastern Taiwan.

In this study, the theoretical age of Japanese glass eels for each arrival batch was estimated based on the time differences between the new moon of the presumptive spawning time and the peak catch time of each glass eel wave in the Yilan estuary, based on data from the Taiwan Japanese Glass Eel Reporting System and the daily fax from the Japan Aquaculture Information News. The arrival waves in Taiwan were found between November and March, corresponding to their spawning times between May and September, with most waves occurring in December and January, corresponding to their spawning times in June and July. The estimated age of each arrival wave in Yilan, Taiwan, were significantly positively correlated with their mean TLs (R^2^ = 0.31, *p* = 0.002). Furthermore, the average estimated larval age was 163.64 ± 6.7 days. The maximum was 179 days and the minimum was 150 days, which showed approximately 29 days difference. These extreme values and differences between them might have reflected the influence of the ENSO events (Fig. 3).

### The mean TLs of specimens in each month in eastern Taiwan

**Figure 4:**
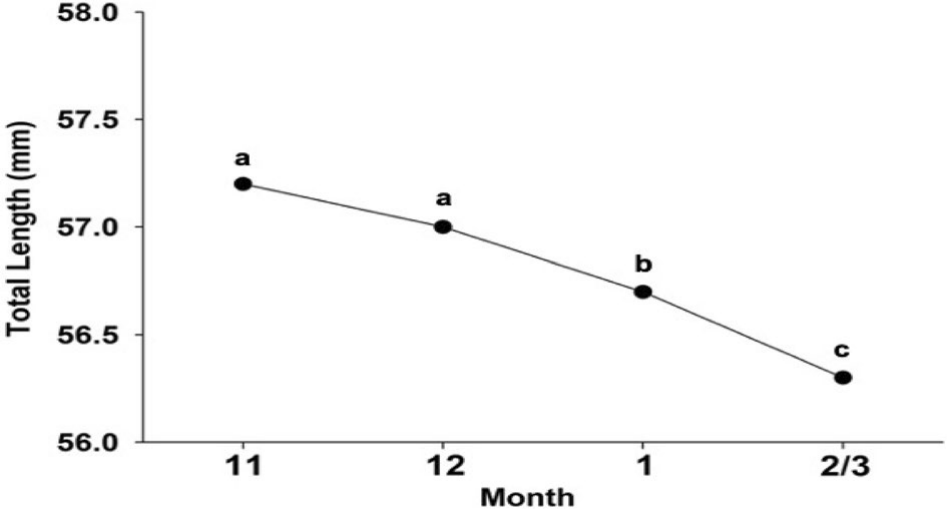
The mean total length of Japanese glass eels from Taiwan (November to February/March).

The mean TLs of the specimens from each month (November to February/March) in eastern Taiwan were: November—57.2 ± 2.1 mm (s.d.), n = 1818; December—57.0 ± 2.4 mm (s.d.), n = 1610; January—56.6 ± 2.3 mm (s.d.), n = 1626; and February/March—56.3 ± 2.1 mm (s.d.), n = 541 (Fig. 4). The mean TLs of Japanese glass eels decreased monthly with significance, with the largest mean TLs in November and December (*p* = 0.198) (early recruits) and lowest mean TLs in February and March (*p* = 0.025) (late recruits).

### The NEC/Kuroshio long-term monthly velocity

According to the previous study, the surface climatological NEC bifurcation latitude is approximately 12.7° N based on 40 years of Simple Ocean Data Assimilation (SODA) reanalysis^36^. The bifurcation latitude moves toward the north with depth owing to the baroclinity of the NEC. The climatological mean of the subsurface (96 m) NEC bifurcation latitude was estimated to be approximately 13.6° N by Meng et al.^36^, which is similar to the estimation of 14° N using the 25-yr HYCOM reanalysis in this study.

**Figure 5:**
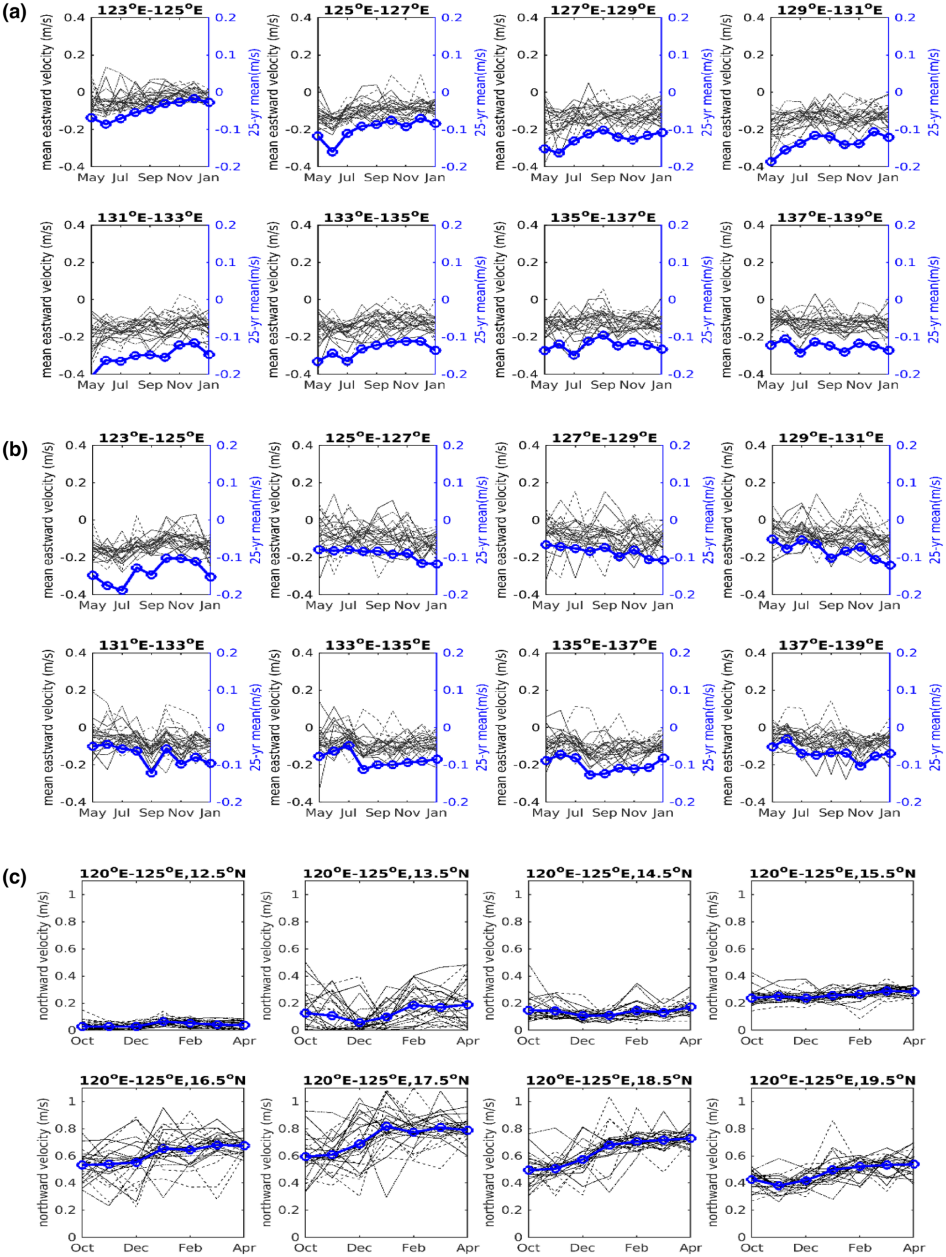
The main stream velocity (m/s, thin lines) of the NEC over 1994–2019 in the region of (**a**) 13–14° N, (**b**) 15–16° N, and (**c**) Kuroshio. The 25-year monthly mean (1994/5–2019/4) is superimposed (blue lines with circles). The enlarged y-axis at the right shows the trend change.

Figure 5a shows the modeled monthly variation of the main stream speed between 13 and 14° N at 100 m depth, corresponding to the south of the NEC bifurcation. Between 135 and 140° E, the monthly change in the main stream speed is unclear. To the west of 135° E, there is a decreasing trend from early summer (May to June) to winter (November to December). This is related to the seasonal movement of NEC bifurcation. From summer to autumn, the NEC bifurcation moves northward; therefore, the flow speed in Fig. 5a decreases with time as the NEC main stream center (maxima) moves northward, away from the region at 13–14° N. Figure 5b shows the monthly variation of the main stream speed between 15 and 16° N, corresponding to area north of the NEC bifurcation. The main stream speed changes in Fig. 5b show the opposite trend of the monthly variation compared to those in Fig. 5a. As the NEC moves northward after May, its main stream center moves closer to the north of the bifurcation (15–16° N), and the flow speed between 15 and 16° N increases correspondingly. Figure 5c shows the monthly variation of the northward Kuroshio (120–125° E). From October to April, the current speed between 15.5 and 19.5° N consistently increases as the NEC bifurcation moves southward. This can accelerate the reach of the Japanese eel to the western North Pacific. To the south of 15° N, a seasonal trend was absent. However, we note that strong nonlinear interaction occurred when the mesoscale collides with the Kuroshio Current, and the effects are difficult to remove. As a result, the interannual variations in Fig. 5c are still large, especially the sections between 16.5 and 18.5° N.

### Numerical simulation of larval transport

**Figure 6:**
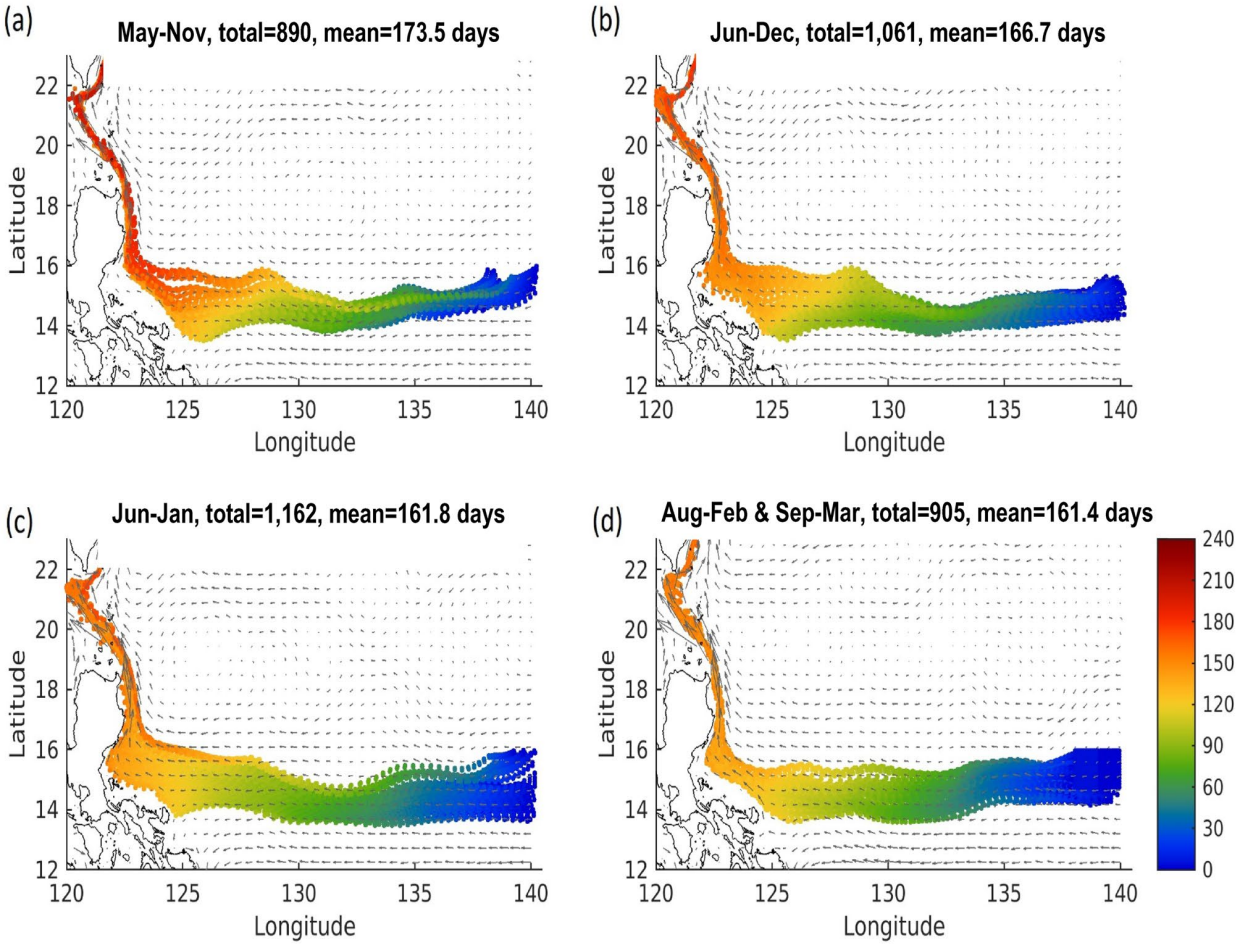
(**a**) The simulated transport pathways and times (color shaded) of larval Japanese eels from the spawning site (137–140° E, 14–16° N) to Luzon Strait released from May 1st. (**b**–**d**) released from June 1st, July 1st, and August/September 1st, respectively.

Several representative tracer simulation experiments further confirmed that the tracers (larvae) drifted along the main stream of the westward-flowing NEC and reached the east coast of the Philippines 3 to 4 months later. They then flowed along the northward Kuroshio and finally approached the south of Taiwan (Fig. 1). The modelled mean tracer drift time during 1994–2019 from the spawning site to the south of Taiwan is presented in Fig. 6. The tracers were released at the beginning of May, June, July, and August/September. The particles released from May 1st required the longest drifting time (173.5 ± 34.6 day), followed by June 1st (166.7 ± 24.2 day), July 1st (161.8 ± 24.2 day), and August 1st/September 1st (160.4 ± 24.2 day) (Fig. 6). The actual drifting days from the spawning area are shaded in Fig. 6. These results are consistent with the monthly flow speed variations shown in Fig. 5. To the north of the NEC bifurcation, at approximately 14° N, the main stream speed increases from early summer to autumn, while the Kuroshio near the Philippine coast further strengthens after autumn. When the particles were released in May, they reached the south of Taiwan (Luzon Strait) between October and December. When the particles were released in August or September, they reach south of Taiwan in a shorter time.

### The monthly catch percentage of the glass eel in Taiwan and Japan

**Figure 7:**
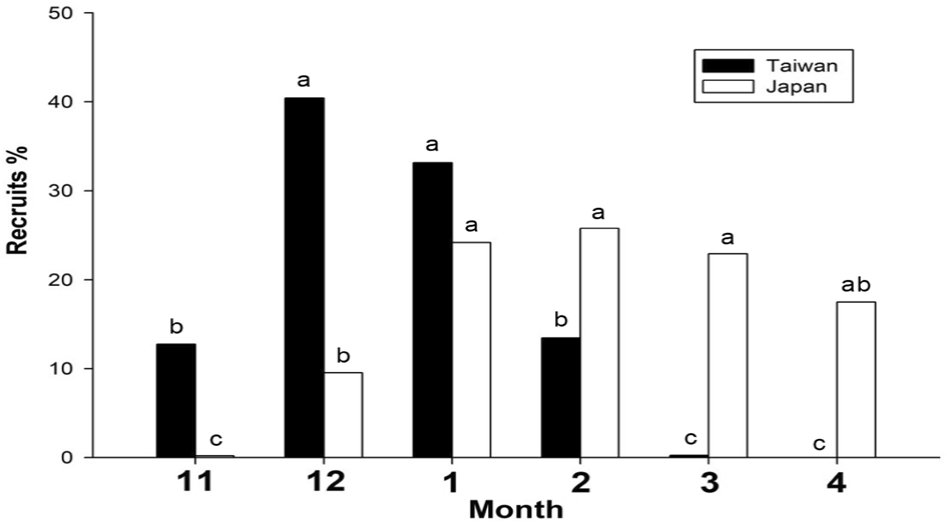
The monthly recruitment percentage of the Japanese glass eel in Taiwan and Japan from November to April.

The recruitment of the Japanese glass eel was mainly between November and February in Taiwan, with an arrival peak in or near December (Fig. 7). There were very few recruits in March in Taiwan. In contrast, the recruitment of the Japanese glass eel usually began in December and ended in late April in Japan, with an arrival peak in or near February (Fig. 7). Furthermore, Table 1 showed the yearly catch data (in percentage) of the time series and the calculated time lag during November to next April in Taiwan and Japan. The highest percentage of monthly recruitments in the period was chosen from both areas to calculate the time lag between Taiwan and Japan. The results showed that in an 11-years span, a 1-month time lag is the largest proportion (55%), In addition, the recruitment lag between Taiwan and Japan was approximately 1.36 months.

**Table 1:** The time series and the calculated time lag of catch data (percentage) during November to next April in Taiwan and Japan.

Location	Taiwan						Japan						Time lag (month)
Month	Nov.	Dec.	Jan.	Feb.	Mar.	Apr.	Nov.	Dec.	Jan.	Feb.	Mar.	Apr.	
2010	0.40	1.20	**1.80**	0.80	0.00	0.00	0.00	0.50	1.20	**4.00**	2.10	1.20	1
2011	0.50	**0.90**	0.30	0.20	0.00	0.00	0.02	0.78	1.11	1.79	2.42	**2.88**	4
2012	0.10	0.30	**0.80**	0.30	0.00	0.00	0.00	0.06	0.50	**2.30**	1.15	1.10	1
2013	2.00	**4.50**	2.50	1.00	0.00	0.00	0.10	1.89	1.75	4.26	**5.10**	4.20	3
2014	0.20	0.30	**0.50**	0.20	0.00	0.00	0.00	2.00	**5.80**	2.82	2.38	1.10	0
2015	0.38	0.72	**1.23**	0.17	0.00	0.00	0.00	0.80	3.00	**5.18**	3.12	1.90	1
2016	1.15	**2.67**	0.48	0.20	0.00	0.00	0.10	1.80	**5.95**	4.00	3.75	2.40	1
2017	0.03	0.07	0.30	**0.60**	0.10	0.00	0.00	0.08	0.44	0.56	**3.86**	**3.86**	1
2018	0.12	**1.45**	0.98	0.20	0.00	0.00	0.00	0.30	0.70	**1.20**	0.90	0.40	2
2019	0.11	**3.61**	2.15	1.13	0.00	0.00	0.00	3.00	**8.00**	5.00	3.00	2.00	1
2020	0.41	1.40	**3.00**	0.90	0.00	0.00	0.01	1.27	**3.16**	2.63	2.17	1.89	0
Mean	0.49	1.56	1.28	0.52	0.01	0.00	0.02	1.13	2.87	3.07	2.72	2.09	1.36

The numbers in bold represent the month with the highest percentage during the period.

### The monthly recruitment proportion between Taiwan and Japan

**Figure 8:**
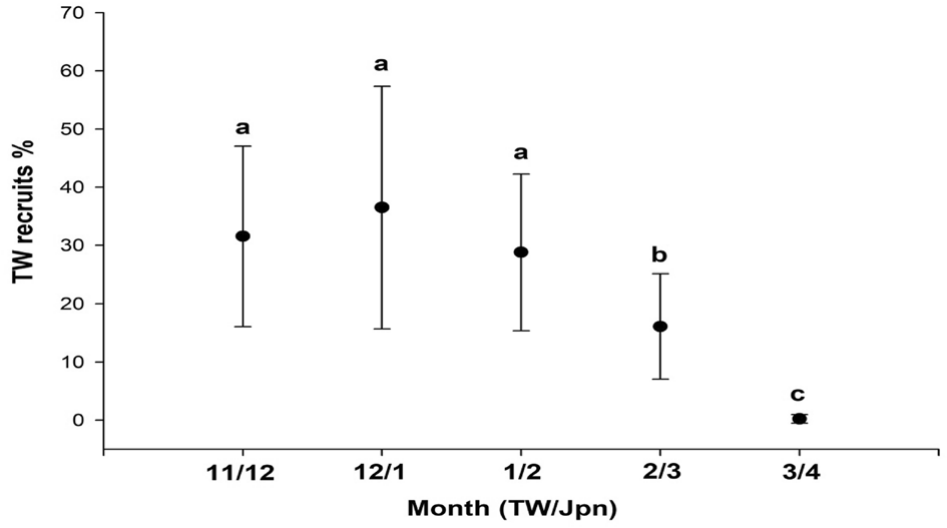
The relative abundance of Japanese glass eels between Taiwan (TW) and Japan (Jpn) from November/December to March/April.

The monthly catch data of the Japanese glass eels in Taiwan and Japan between 2010 and 2021 are shown in Supplementary Table S1. Since the movement of recruitment waves of the Japanese glass eel from Taiwan to Japan requires approximately 1–1.5 months with a similar catch pattern, which could further assume that the glass eels caught in November in Taiwan and December in Japan were mainly from the same cohorts. Thus, they were used to calculate the relative proportion of glass eel recruitment in Taiwan for each month. The results indicate that the relative percentage of glass eels in Taiwan was significantly elevated in early recruitment months (November–January), then decreased in late recruitment months (February–March) (*p* = 0.693 in 11/2 and 12/1, *p* = 0.393 in 12/1 and 1/2; *p* = 0.023 in 1/2 and 2/3; *p* < 0.001 in 2/3 and 3/4; *p* < 0.001 in 3/4 and 11/12) (Fig. 8).

## Discussion

In this study, the Japanese glass eel specimens were collected at night during the fishing season and mainly using a boat net in Taiwan. Therefore, the catch was less affected by the tidal variations. A recent study demonstrated the probability density distributions of total length based on a cohort of eels captured in the Yilan River, Taiwan from 2010 to 2019, and the arrival waves seem more likely related to the New Moon hypothesis. It is worth to be noted that the “waves” could be seen in eastern Taiwan, especially Yilan since it is the first place to receive recruitment waves of *A. japonica*. Therefore, the individual variation in the daily age (and the TL as well) should be lower in Yilan, and the dynamics of glass eel arrivals in the Yilan offshore area may serve as a proper assessment of monthly larval cohorts from the spawning ground. According to the analysis results in this study, the estimated age of each arrival wave in Yilan was found to positively correlate with the mean TL (Fig. 3), supporting the relationship between age and TL of the glass eel. The varied mean TL of each arrival batch in Taiwan suggested that each wave possessed a different mean transport time. Variations in mean ages between arrival waves may reflect differentiation in the current speed during larval transport from the spawning site to Taiwan. Alternatively, these batches of glass eels may spawn at different sites in the range of 12–16° N during the new moon period and then drift along different routes on the NEC and Kuroshio to the Yilan offshore area. Han et al.^21^ found that each batch of glass eels caught offshore in December of three consecutive year classes (2010–2012) at Yilan, had a narrow range of ages between years according to the daily increments analysis of the otoliths. This implies that most individuals in the arrival peaks spawn in the same month and thus belong to the same cohort. Furthermore, tracer simulation also supported that each batch of glass eels takes approximately 160–170 days to reach the Luzon Strait from their spawning site (Fig. 6). All of this data suggests that the potential mixing of glass eels between monthly spawning cohorts was low, at least in Taiwan.

According to the information from Japan Aquaculture Information News, the arrival waves of the glass eel to Japanese coastal areas also happened monthly, especially noticeable in the first 2 months of the fishing season. Although few eels may stay longer in estuaries in Japan due to the low water temperature or escaping from caught in previous month from March to April. However, the glass eel could perform upstream migration with water temperature higher than 8 °C, and the mean water temperature along the pacific coast of the Japan is higher than 8 °C^5,47^. Thus, the water temperature seems not an important factor. In addition, it is known that the glass eels in Japan were caught by the hand-trawling net or a fyke net in the estuaries. On the other hand, the glass eels in Taiwan were caught by the hand-trawling net, a fyke net and a boat net (main force) in offshore areas. Therefore, it would indeed increase the possibility with a mixing up of the new arrival and old eels of the captured glass eels in Japan, especially for the late recruits, although the ratio of the new arrival eels seems still accounted for most of the proportion. Furthermore, the glass eel recruiting to Taiwan and Japan had a similar catch pattern. Therefore, it was reasonable to assume that the arrival waves with approximately 1 month of time lag between Taiwan and Japan may mostly the same cohort.

One of the main dispersal routes of Japanese eel larvae is from Taiwan to Japan by the Kuroshio stream. It is estimated that the time needed for the larval eels to travel from Taiwan to Kyushu, Japan (approximately 1000 km) is 25.3 ± 6.4 days^15^. The mean velocity of the Kuroshio along the Pacific coast of Japan is approximately 0.7–1.4 m/s^48,49^; thus, it takes 8–16 days for the eel larvae to be transported from western to eastern Japan (approximately 1000 km). This means that the recruitment time lag between Taiwan and Japan is expected to be close to one month, and the mean TLs of the glass eels in Japan should be greater than those in Taiwan because of their increased mean ages. According to the previous study, although the states at measurement were different, the mean TLs of the glass eels in Japan ranged between 57.9 ± 2.0 (s.d.) and 60.1 ± 2.3 (s.d.) mm, which is greater than that of the glass eel in Taiwan (56.9 ± 2.0 (s.d.) mm)^31^. Furthermore, the TLs measurement in our laboratory under the same condition (measured after one month of storage in 95% ethanol) also showed the mean TLs of the glass eels in Japan was greater than that of glass eel in Taiwan with significance (*p* < 0.05). The results of the positive correlation between the estimated age and the total length of the glass eel recruiting to eastern Taiwan (Yilan and Hualien) (Fig. 3) also support the relationship between larval age and the total age of the glass eel logically.

In addition, the recruitment of the Japanese glass eel usually started in November in Taiwan and December in Japan, and the arrival peak of the Japanese glass eel was December/January in Taiwan and January/February in Japan (Fig. 7), coinciding with a lag of approximately 1 month, which implied that the mean TLs of the glass eels in Japan are expected to be larger than those of glass eels in Taiwan. The TL analysis of the glass eels in this study also showed that the TL of the individuals captured from Japan were significantly larger than those captured from Taiwan.

Japanese eels spawn in restricted areas (12–16° N and 141–142° E) during a specific time span (May through September)^15,50^. The larvae are passively transported via the NEC to the east Philippines for 3–4 months and then enter the Kuroshio and move toward their habitats in East Asia. For the glass eels that arrived in Taiwan, the dispersal distance was longer in the NEC (approximately 2500 km) than in the Kuroshio (closer to 1000 km), and the mean NEC velocity was generally low compared to that of the Kuroshio (Fig. 5). However, the monthly velocity variations of the NEC were low compared with those of the Kuroshio (Fig. 5). A previous study has also demonstrated that recruitment dynamics and distribution of Japanese glass eels might change with global warming, due to changes in the velocity, structure, and location of the NEC, NEC bifurcation, and Kuroshio with warming climate^51^. This suggests that the transport time the glass eels need to reach the Luzon Strait from the spawning site is significantly impacted by both the NEC and the Kuroshio. Eel larvae that spawn between May and June (early recruits) require a longer transport time (167–174 days) than those spawning between August and September (late recruits, 160 days) (Fig. 6). Therefore, a greater percentage of early recruits may metamorphose in the southern areas of East Asia due to their slower dispersal. By contrast, more late recruit eel larvae may metamorphose in the northern areas of East Asia due to their faster transport. Indeed, the relative abundance of glass eels near Taiwan relative to Japan was significantly higher in earlier recruitment months (November–December), and significantly lower during later recruitment months (February–March) (Fig. 8). Seasonal variations in the location of the NEC bifurcation can be entertained as one of the most important factors, since it is located at its southernmost latitude in May, and moves northward after that, reaching its northernmost point in September. In other words, the distance between the NEC bifurcation and Kuroshio might be wider when the NEC bifurcation is in the southern region of its range, thereby causing a longer duration of drift among the earlier recruitment cohort. In March, Japanese glass eels were scarce in Taiwan; however, they were abundant in Japan in April. This supports the concept that late recruits, which were transported with faster oceanic current velocity, would bypass Taiwan and metamorphose mainly downstream of the Kuroshio and then continue on to Japan.

In terms of the interannual variation, in the tropical and subtropical Pacific, the El Niño-Southern Oscillation (ENSO) mostly dominates the interannual variability while the ocean dynamics and thermal conditions are significantly changed with ENSO phases. The NEC can response to ENSO-related atmospheric forcing but the relationship is not rigorous^52^. To investigate the influence of ENSO phases on the travel time of Japanese eels, we further perform additional particle tracking experiments under different ENSO phases. The composite ocean conditions are redefined as El Niño, La Niña, and Neutral (Table 2) based on the Oceanic Niño Index (ONI) (https://ggweather.com/enso/oni.htm). The simulation period of the spawning is from May to September, which is often in the decaying and developing phase of the ENSO years. To avoid complexity, only developing phase of moderate, strong and very strong ENSO years were selected, and the rest are defined as “Neutral or weak ENSO”. Previous research has reported an anomalous northward/southward shift of the location of the NEC bifurcation during an El Niño/La Niña developing and mature stage. Our particle tracking results released from July to September during the ENSO years is consistent with the NEC bifurcation shifts. During La Niña, the particle trajectories are further south compared to El Niño. However, the results released from May and June do not show the corresponding shifts of the trajectories. In general, our climatological analysis is more consistent with the neutral/weak ENSO condition.

**Table 2:** Mean glass eel travel time for different ENSO conditions.

Type of the ENSO phase	El Niño	La Niña	Neutral or weak ENSO
Composite years	1994, 1997, 2002, 2009, 2015	1995, 1998, 1999, 2007, 2010, 2011	1996, 2000, 2001, 2003, 2004, 2005, 2006, 2008, 2012, 2013, 2014, 2016, 2017, 2018
Mean travel time (days) (starting from May)	164.7	140.8	192.1
Mean travel time (days) (starting from June)	128.2	155	184.1
Mean travel time (days) (starting from July)	138.8	170.7	172.6
Mean travel time (days) (starting from Aug and Sept)	139.2	160.3	158.8

The location of NEC bifurcation varies both seasonally and interannually. The annual excursion of the NEC bifurcation, about 2° latitude^32^, is at a similar scale to the ENSO-related anomaly, which brought complexity for the traveling time during ENSO years. Generally, the traveling time is shorter during El Niño developing stage, except for those tracking starting from May. However, the connection between NEC and ENSO is more complicated than what described here. Different types of ENSO (i.e. the canonical El Niño, the Central Pacific El Niño I, and the Central Pacific El Niño II^53^) results in different NECs, and the detailed investigation of the ENSO influence on the NEC so as to the glass eels’ travel time and body length is beyond the scope of this study.

Although the mean TLs of Japanese glass eels in Taiwan decreased monthly with significance, the mean TLs of each batch ranged between 54.6 and 59.9 mm with high variation. Multiple biotic/abiotic factors must be involved in this TL variation. In a previous study, it was found that ENSO events had a substantial impact on the TL of glass eels in Taiwan^26^. The TLs of Japanese glass eels were significantly greater during El Niño years and less during La Niña years. The northward shifts in the NEC bifurcation and the southward shift of the salinity front during the El Niño years might have led to larvae encountering slower currents and broadening the distance between the spawning site (south of the salinity front) and the NEC bifurcation^14,54,55^, thereby extending the journey time required for the larvae to enter the Kuroshio from their spawning grounds^26^. Since ENSO events occur periodically, the effect of ENSO on the transport time of eel larvae in each month would be offset over the long term.

It has been suggested that local eddy currents along the transport route may trap some leptocephali and result in a small amount of mixing between monthly cohorts^34^. Chang et al.^56^ indicated that v-larvae may be able to remain in eddies passively (physical trapping) due to mesoscale eddy nonlinearity, and/or actively (biological attraction) due to rich food supplies in those eddies. In addition, otolith analysis indicated that Japanese eels may have a recruitment route through the mesoscale eddies to the east of Taiwan, in addition to the direct transfer route from the NEC to the Kuroshio^31^. However, based on the present study, the transport of each eel recruitment batch was generally stable in Taiwan, suggesting that the simultaneously spawning eel cohort usually forms a group and moves together. The eel larvae transported by eddies, if present, may mainly merge downstream of the Kuroshio, thus reducing the mixing degree between monthly cohorts in Taiwan. In either situation, direct observational fish larvae data from the open ocean in mesoscale eddies is necessary to evaluate their true degree of contribution to eel larvae dispersal.

## Conclusion

Among the East Asian countries, Taiwan is nearest to the spawning ground of the Japanese eel, and is the first place to catch the recruited glass eels up to 1 month earlier than in Japan and China^5,7,15^. The glass eels recruited to Taiwan had the lowest mean larval duration and age when compared with those from other sites^15^. Therefore, the dynamics of the glass eel arrival in Taiwan may serve as a useful indicator of recruitment patterns in other countries^7^. The monthly changes in the NEC and Kuroshio velocity may play an important role in affecting the TL as well as the distribution of the Japanese glass eel, by causing more abundance in Taiwan for the early recruits and in Japan for the late recruits.

## Supplementary Information

Supplementary Table S1.

## Data Availability

The datasets generated during and/or analyzed during the current study are available from the corresponding author on reasonable request.
