# Supplementary material for: North Equatorial Current and Kuroshio velocity variations affect body length and distribution of the Japanese eel Anguilla japonica in Taiwan and Japan
Source: Sci Rep. 2022 Feb 21;12:2888. doi: 10.1038/s41598-022-06669-8 (PMC8861004; doi:10.1038/s41598-022-06669-8)
Supplement: Supplementary file 1 — Supplementary Table S1. [file 41598_2022_6669_MOESM1_ESM.docx]

Supplementary Table S1. Monthly catch of Japanese glass eels in Taiwan (TW) and Japan (JPN) between 2010 and 2021

| Year | 2010–2011 | | 2011–2012 | | 2012–2013 | | 2013–2014 | | 2014–2015 | | 2015–2016 | | 2016–2017 | | 2017–2018 | | 2018–2019 | | 2019–2020 | | 2020–2021 | |
| --- | --- | --- | --- | --- | --- | --- | --- | --- | --- | --- | --- | --- | --- | --- | --- | --- | --- | --- | --- | --- | --- | --- |
| Month | JPN | TW | JPN | TW | JPN | TW | JPN | TW | JPN | TW | JPN | TW | JPN | TW | JPN | TW | JPN | TW | JPN | TW | JPN | TW |
| Nov. | 0 | 0.4 | 0 | 0.5 | 0 | 0.1 | 0.1 | 2.0 | 0 | 0.2 | 0.0 | 0.4 | 0.1 | 1.2 | 0 | 0.0 | 0 | 0.1 | 0 | 0.1 | 0 | 0.4 |
| Dec. | 0.5 | 1.2 | 0.8 | 0.9 | 0.1 | 0.3 | 1.9 | 4.5 | 2.0 | 0.3 | 0.8 | 0.7 | 1.8 | 2.7 | 0.1 | 0.1 | 0.3 | 1.5 | 3.0 | 3.6 | 1.3 | 1.4 |
| Jan. | 1.2 | 1.8 | 1.1 | 0.3 | 0.5 | 0.8 | 1.8 | 2.5 | 5.8 | 0.5 | 3.0 | 1.2 | 6.0 | 0.5 | 0.4 | 0.3 | 0.7 | 1.0 | 8.0 | 2.2 | 3.2 | 3.0 |
| Feb. | 4.0 | 0.8 | 1.8 | 0.2 | 2.3 | 0.3 | 4.3 | 1 | 2.8 | 0.2 | 5.2 | 0.2 | 4.0 | 0.2 | 0.6 | 0.6 | 1.2 | 0.2 | 5.0 | 1.1 | 2.6 | 0.9 |
| Mar. | 2.1 | 0 | 2.4 | 0 | 1.2 | 0 | 5.1 | 0 | 2.4 | 0 | 3.1 | 0 | 3.8 | 0 | 3.9 | 0.1 | 0.9 | 0 | 3.0 | 0 | 2.2 | 0 |
| Apr. | 1.2 | 0 | 2.9 | 0 | 1.1 | 0 | 4.2 | 0 | 1.1 | 0 | 1.9 | 0 | 2.4 | 0 | 3.9 | 0 | 0.4 | 0 | 2.0 | 0 | 1.9 | 0 |
